# Depth of slab breakoff in Archean: the role of radiogenic heat production of continental crust and eclogitization of oceanic crust

**DOI:** 10.1038/s41598-025-29212-x

**Published:** 2026-01-03

**Authors:** Yin-Long Fan, Li-Fei Zhang, Yang Wang, Zhong-Hai Li

**Affiliations:** 1 SKLab-DeepMinE, MOEKLab-OBCE, School of Earth and Space Sciences, Peking University, Beijing, China; 2 State Key Laboratory of Earth System Numerical Modeling and Application, College of Earth and Planetary Sciences, University of Chinese Academy of Sciences, Beijing, China

**Keywords:** Slab breakoff, Radiogenic heat production, Eclogitization, Archean collisional orogens, Numerical modeling, Planetary science, Solid Earth sciences

## Abstract

The slab breakoff has been applied to many collisional orogen around the world to explain postcollisional magmatism and metamorphism. For Phanerozoic orogens, multiple lines of evidence indicate that ultrahigh-pressure metamorphic rocks are exhumed from depths of 100–400 km, implying deeper slab breakoff depths. However, the slab breakoff depth in Archean orogens is less certain, and the factors governing the depth of slab breakoff remain poorly constrained. In this study, we use a 2D numerical model to identify the key parameters controlling slab breakoff and to quantitatively evaluate the depth of slab breakoff. The results indicate that a high degree of eclogization of oceanic crust, fast convergence rates, and low radiogenic heat production of continental crust generally promote slab breakoff at greater depths. Otherwise, the shallow slab breakoff mode is favored. For Archean collisional orogens, it is more likely for shallow breakoff to occur, due to the higher radiogenic heat production and mantle potential temperature, potentially explaining the rare occurrence of ultra-high pressure metamorphic rocks in the Archean. We also quantified the effects of other factors, including the density of subcontinental lithospheric mantle and age of oceanic lithosphere, which play secondary roles in determining slab breakoff depth.

**Supplementary Information:**

The online version contains supplementary material available at 10.1038/s41598-025-29212-x.

## Introduction

**Fig. 1:**
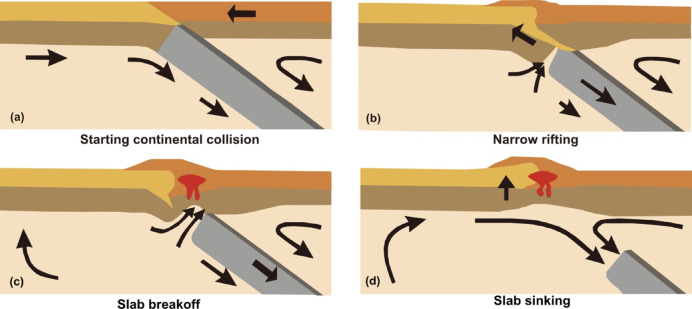
A schematic illustration of the slab breakoff model (modified from Davies and von Blanckenburg^2^).

Continental collision normally follows oceanic subduction under the convergent forces of lateral “ridge push”, oceanic “slab pull”, and/or mantle convective flow^1^. A remarkable event during continental collision is the slab breakoff or slab detachment, which was first proposed by Davies and von Blanckenburg (1995)^2^. Slab breakoff occurs when buoyant continental lithosphere follows denser oceanic lithosphere into the subduction zone. The buoyancy of the subducted continental lithosphere opposes the negative buoyancy of the subducted oceanic lithosphere, ultimately leading to breakoff and detachment of the preceding oceanic slab (Fig. 1).

Slab breakoff has been widely recognized as a key geodynamic process and has been invoked in the interpretation of different geological phenomena in many continental collisional orogens^2,3^, although the slab retreat may potentially modify the thermal structure of orogens^4,5^. This model is substantiated by a range of geophysical observations in the Alpine-Himalayan orogenic system, such as the Western Alps orogen, the Eastern Alps orogen^6–8^, Anatolia-Zagros orogen^9,10^, and Himalaya orogen^11,12^, derived from various geophysical approaches. On the other hand, the model has been supported by diverse geological observations, including systematic geochemical evolution of post-collisional magmas^13,14^, rapid topographic uplift linked to slab detachment^15^, and ore mineralization processes potentially driven by breakoff-induced asthenosphere upwelling^16^.

The depths of slab breakoff have been investigated extensively by analytical and numerical models, and it has been suggested that breakoff could occur at depths ranging from 40 to 400 km^17–24^. Previous studies have primarily focused on the influence of rheological properties of oceanic plates, or lithospheric thickness, and convergence velocity on the breakoff depth^20,22^. Depths of slab breakoff may be fairly well constrained for Phanerozoic orogens, but the slab breakoff depth of Archean orogens remains unclear. There are many changes in both metamorphic and magmatic signatures in the geological record of the Archean to Proterozoic transition, representing a major change in the dynamics of collisional orogens^25^. It is worth noting that Archean continental and oceanic plates differed significantly from those of the modern Earth, and their potential influence on slab breakoff depths is not well-constrained.

It is widely accepted that the Earth has been cooling since its formation due to the decline in heat producing elements. As the continental crust serves as an major reservoir of heat-producing elements, its thermal structure has undergone substantial evolution since the Archean^26^. Global geochemical analyses of continental crust rocks reveal considerable variability in the heat production rate, ranging from 1 to 4 μW m^−3^, largely attributed to complex differentiation and redistribution processes^24–28^. Given that the concentration of heat-producing elements in the crust is affected by radioactive decay, the back-calculations from present-day values suggest that the average heat production rates were approximately 1.5 to 1.75 times higher in the Archean^29–31^. In any case, the early Earth was potentially characterized by a much higher Moho temperature owing to the initial heat produced by the Earth accretion-differentiation and to a more intense decay of heat producing elements^29^. The concentration variations of heat-producing elements within the upper crust exert a significant influence on the thermal structure and mechanical strength of continental lithosphere^32^. Compared to the continental crust, the influence of heat-producing elements in the oceanic crust on plate thermal structure is significantly less pronounced. However, eclogitization of oceanic crust has been proposed as the significant contributor to slab pull forces in subduction zones^33^. Experimental studies demonstrated that the oceanic-derived eclogites can undergo up to a 16% increase in density beyond the depths of 60-80 km^34,35^. Field observations show that this reaction is strongly relevant to fluid availability, with dry rocks often exhibiting incomplete or inhibited transformation under typical eclogitization pressure-temperature conditions^36^. To date, no systematic investigation has quantitatively evaluated how continental crust composition changes and phase transition degree affect the depth of slab breakoff.

In this study, we constructed 2-D thermo-mechanical models to systematically investigate key parameters for the slab breakoff dynamics. Our results indicate that the depth of slab breakoff is primarily controlled by the convergence rate, radiogenic heat production of felsic crust, and eclogitization degree of preceding subducting oceanic crust. In contrast, the age of oceanic lithosphere and depletion degree of subcontinental lithospheric mantle exert negligible influence on slab breakoff depth.

## Methods and model setup

**Fig. 2:**
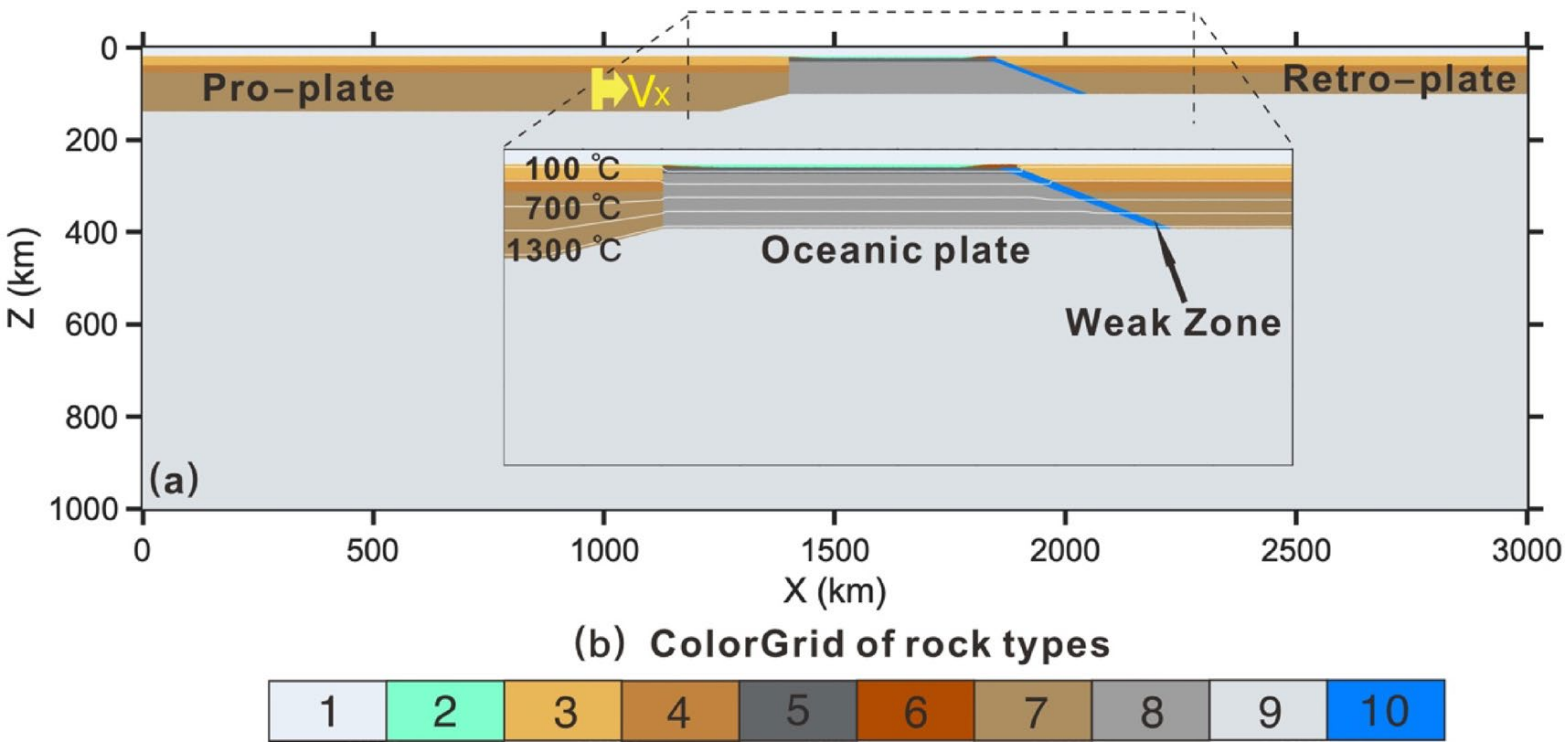
Initial model configuration and boundary conditions. (**a**) The numerical box is 3000 × 1000 km, with isotherms (white lines) plotted from 100 °C at intervals of 200 °C. A constant velocity of 5 cm/yr is assigned within the proplate. (**b**) The colored grid illustrates different rock types: 1 = air; 2 = water; 3 = felsic upper crust; 4 = mafic lower crust; 5 = oceanic crust; 6 = sediment 7 = lithospheric mantle of pro- and retro-plate; 8 = oceanic lithospheric mantle; 9 = asthenosphere; 10 = initial weak zone. The detailed properties of different rock types are provided in Table S1 and S2. The software MATLAB (R2024a, https://www.mathworks.com/products.html) was used to create the figure.

We use a 2D thermo-mechanical code I2VIS^37^ based on finite-difference and marker-in-cell techniques to solve the mass, momentum, and energy conservation equations (see details in supplementary information). The size of the model domain is 3000 km $\times$ 1000 km (Fig. 2) and it is resolved with a regular rectangular grid of 1500 (horizontal) $\times$ 500 (vertical) nodes, containing 6 million randomly distributed markers.

The initial model configuration comprises two continental lithospheric plates separated by a 500-km-wide oceanic lithospheric segment, with a rheologically weak zone to initiate subduction. The continental lithosphere is composed of a 35-km-thick crust, which is further subdivided into a felsic upper crust (20 km) and a mafic lower crust (15 km), underlain by an 85-km-thick mantle. The flow law of “wet quartzite” is used for the felsic crust, “Plagioclase An75” for the lower continental crust, and “dry Olivine” for the lithospheric mantle and the asthenosphere, as generally used for a continental lithosphere. A lower plastic effective internal friction angle (sin(ϕ) = 0.006) is used for the weak zone. The initial continental geotherm was computed using a steady-state approximation, with surface temperature fixed at 0 °C and lithosphere-asthenosphere boundary temperature set to 1350 °C. Radiogenic heat production is incorporated within the felsic upper crust to reflect compositional heat source contributions and subsequent model evolution. The oceanic crust is modeled with an 8-km-thick basaltic crust underlain by a 78-km-thick anhydrous peridotite layer. In an additional series of experiments, the thickness of the oceanic crust increases from 8 to 28 km, correlating with mantle potential temperature rising from 1350 °C to 1550 °C. The thermal structure of the oceanic lithosphere is defined using a half-space cooling age ranges from 20 to 140 Ma. Eclogitization of subducted basaltic crust is taken into account by linearly increasing the density of the crust with pressure from 0% to different degree in the P–T region between the experimentally determined garnet-in and plagioclase-out phase transitions in basalt^34^. Each lithology is characterized by visco-plastic rheology and variable thermal conductivity (see Table S1 and S2 in supplementary information). An adiabatic temperature gradient of 0.5 °C/km is applied within the asthenospheric mantle.

The top surface of the lithosphere is implemented as an internal free surface by incorporating an overlying 18-km-thick layer with low viscosity (1 kg/m^3^) and viscosity (10^19^ Pa s). Free-slip velocity boundary conditions are imposed on all boundaries except the permeable lower boundary^38^. The left plate is driven by imposed convergence velocities (Vx) inside the domain which remains fixed relative to the coordinate system.

## Results

We conducted 40 numerical experiments with distinct parameters to quantify controls on slab breakoff depth. The models incorporate the eclogitization of oceanic crust, implemented through a density increase ranging from + 4% to + 16%. For the continental lithosphere, we account for the variation in radiogenic heat production within the felsic crust (1 to 4 μW/m^3^) and compositional density reduction in the subcontinental lithospheric mantle (0 to − 60 kg/m^3^). The age of the oceanic lithosphere remains constant along the plate strike. By varying the age of oceanic plate, we indirectly modulate the mechanical strength of oceanic lithosphere through modification of the initial thermal structure. It was worth noting that the selected parameters do not encompass all potential influence factors, but rather target those most likely to affect the depth of slab breakoff. Systematic parametric tests reveal different depths of slab breakoff and model evolution, with results summarized in Table S3. Based on model results, three endmember modes of slab evolution are identified: (1) deep slab breakoff model (≥ 100 km); (2) shallow slab breakoff model (< 100 km); (3) breakoff failure.

### Deep slab breakoff

**Fig. 3:**
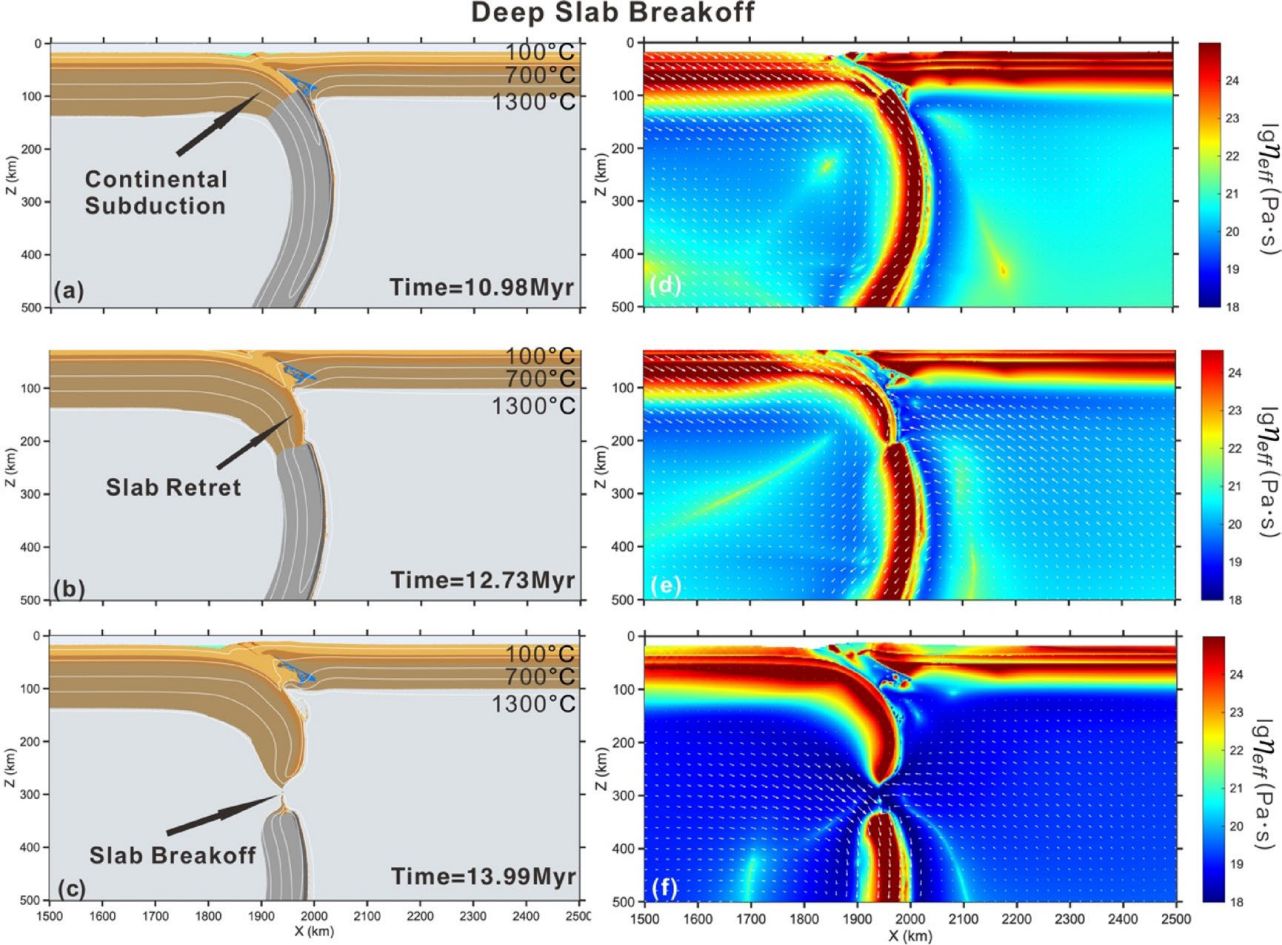
Evolution of deep slab breakoff with lower radiogenic heat production in felsic crust (1 μW/m^3^). Mantle potential temperature is 1350 °C. The cooling age of oceanic lithosphere is 60 Ma. The convergence rate is 5 cm/yr. (**a**)–(**c**) composition and temperature field of model evolution. (**d**)–(**f**) effective viscosity field with velocity vectors of model evolution. Colors of rock types are as in Fig. 2. Time (Myr) of model evolution is given in each panel. The white lines are shown for isotherms, starting from 100 °C at intervals of 300 °C. The software MATLAB (R2024a, https://www.mathworks.com/products.html) was used to create the figure.

This end-member scenario exhibits a deep slab breakoff case (> 100 km) with slab breakoff initiating approximately 4 Myr after the onset of continental collision. In this model, the subducting oceanic plate is driven at a convergence rate of 5 cm/yr. After ocean basin closing the buoyant continental crust is subsequently entrained into the subduction channel, driven by the negative buoyancy of the preceding oceanic slab. The former passive margin stalls at a depth of 100–125 km due to the positive buoyancy of the continental crust (Fig. 3a). This stage is typically characterized by slab decoupling from the overriding plate, concomitant with mantle flow. The temperature along the slab-mantle interface increases due to conductive heat transfer and advective thermal equilibration, which further weakens the continental crust. Eclogitization of the subducted oceanic crust enhances slab pull forces through metamorphic densification, leading to slab necking in ocean-continent transition via viscous deformation (Fig. 3d). Finally, slab breakoff occurs at the depth of approximately 300 km after the initial continental collision (Fig. 3c).

### Shallow slab breakoff

**Fig. 4:**
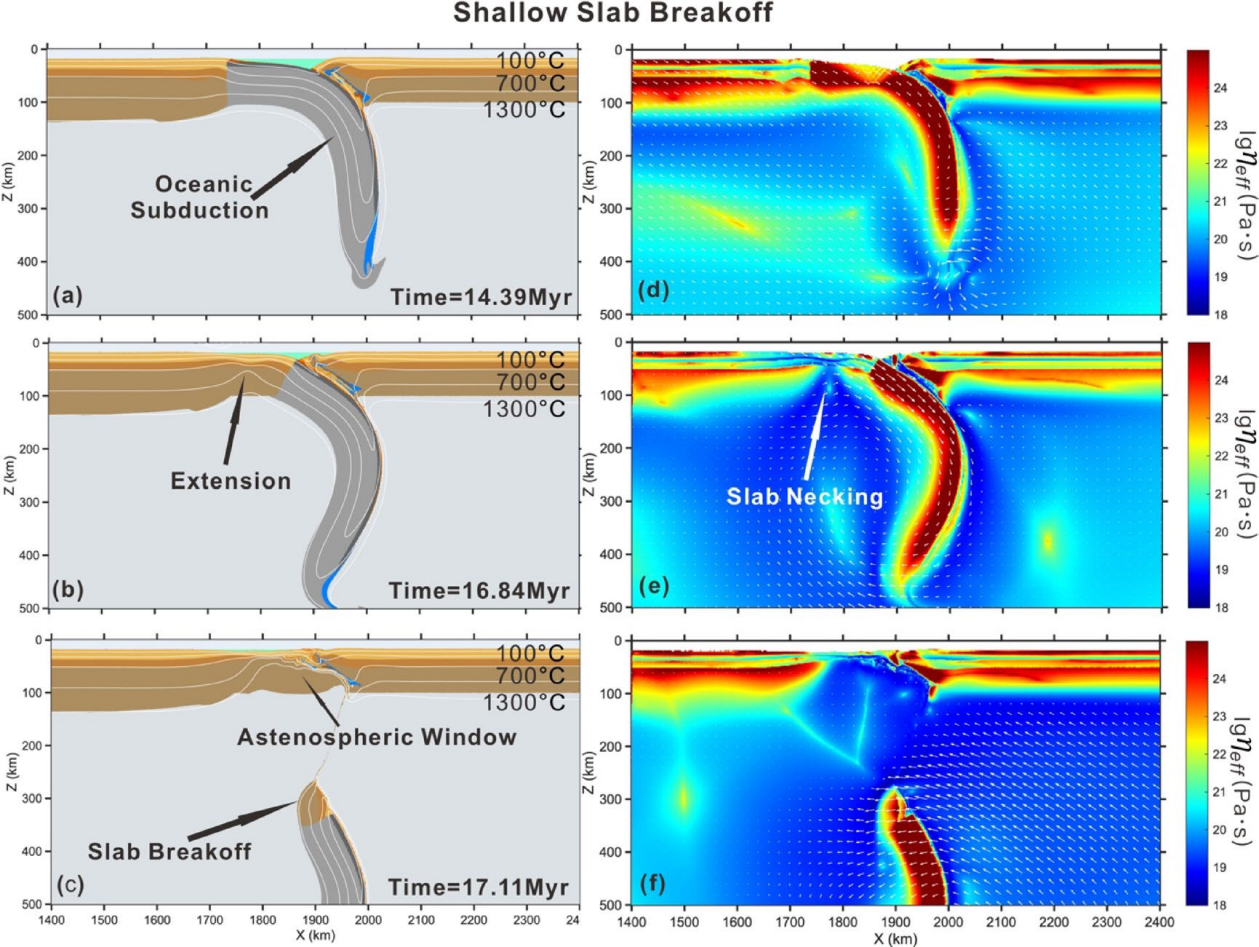
Evolution of shallow slab breakoff with higher radiogenic heat production in felsic crust (4 μW/m^3^). Mantle potential temperature is 1350 °C. The cooling age of oceanic lithosphere is 60 Ma. The convergence rate is 2.5 cm/yr. (**a**)–(**c**) composition and temperature field of model evolution. (**d**)–(**f**) effective viscosity field with velocity vectors of model evolution. Colors of rock types are as in Fig. 2. Time (Myr) of model evolution is given in each panel. The white lines are shown for isotherms, starting from 100 °C at intervals of 300 °C. The software MATLAB (R2024a, https://www.mathworks.com/products.html) was used to create the figure.

Model with high radiogenic heat production (4 μW/m^3^) within felsic crust and slower convergence rates (< 2.5 cm/yr) exhibits distinct behavior, i.e., shallow breakoff (Fig. 4). In this case, the continental crust subduction does not proceed following oceanic subduction. Instead, the high radiogenic heating of felsic crust leads to a hot thermal structure of continental lithosphere and thermally weakens the continental plates. Consequently, the horizontal extension localizes in the buoyant continental lithosphere where the tensile stresses exceed the local yield strength of the slab (Fig. 4b). Simultaneously, the subducting and overriding plate decouple along the subduction channel. A portion of the subcontinental lithospheric mantle is subsequently dragged into the asthenosphere by the subducting oceanic slab. The depth of breakoff is very shallow (approximately 40 km), and rapid slab breakoff takes place soon after the onset of slab necking (approximately 0.3 Myr). A fraction of the continental crust attached to the oceanic lithosphere sinks into deep mantle without exhuming to the surface. Meanwhile, this shallow breakoff leads to mantle upwelling, which causes to the opening of an asthenosphere window and thermally weakens the pro-continental crust.

### Failed slab breakoff

**Fig. 5:**
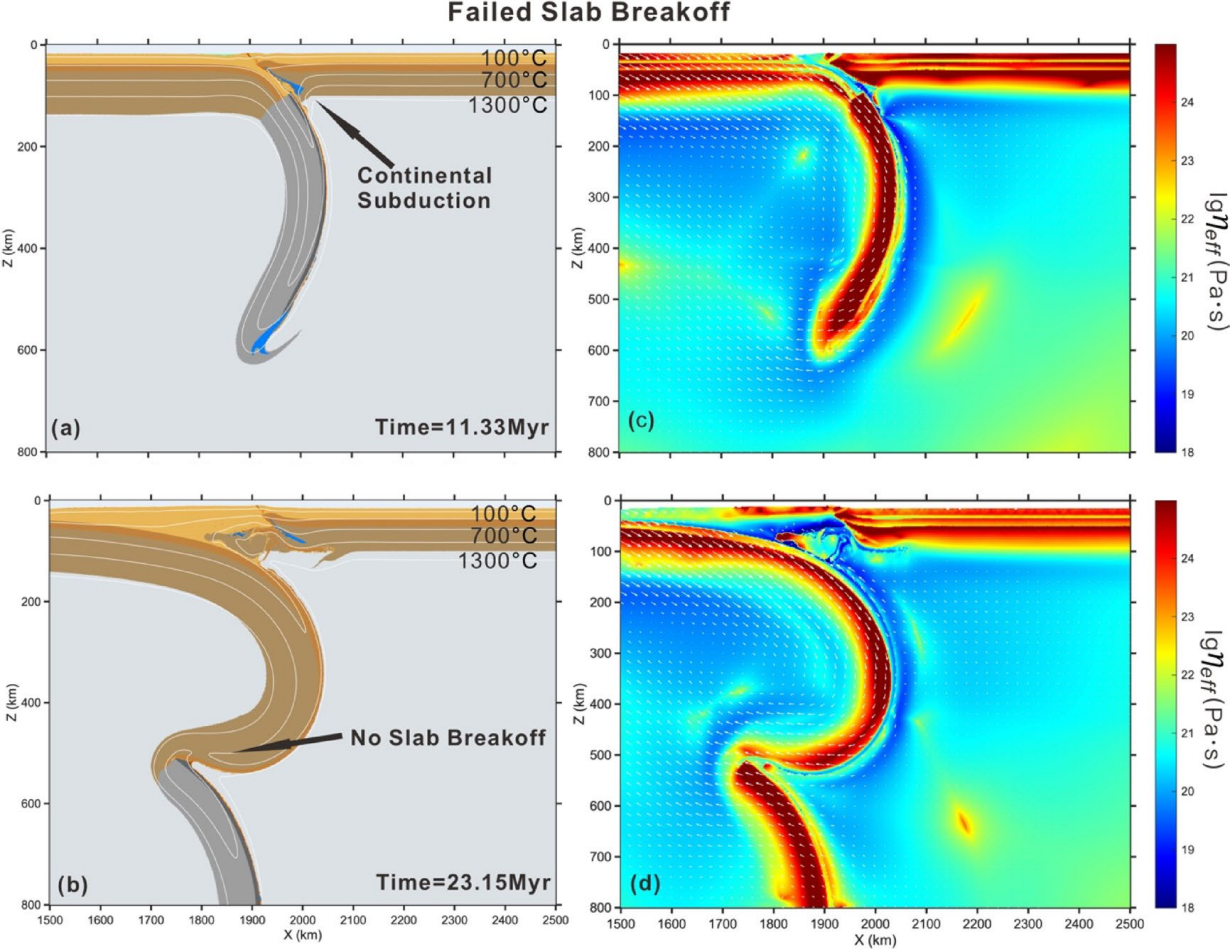
Evolution of failed slab breakoff with lower radiogenic heat production in felsic crust (1 μW/m^3^), as well as fast convergence rate (7.5 cm/yr). Mantle potential temperature is 1350 °C. The cooling age of oceanic lithosphere is 60 Ma. (**a**)–(**c**) composition and temperature field of model evolution. (**d**)–(**f**) effective viscosity field with velocity vectors of model evolution. Colors of rock types are as in Fig. 2. Time (Myr) of model evolution is given in each panel. The white lines are shown for isotherms, starting from 100 °C at intervals of 300 °C. The software MATLAB (R2024a, https://www.mathworks.com/products.html) was used to create the figure.

Under conditions of a rapid convergence rate (7.5 cm/yr) combined with low radiogenic heat production within felsic crust (1 μW/m^3^), the pro-plates subduct continuously into the asthenosphere, and no distinct slab breakoff is predicted (Fig. 5). Similar to the deep breakoff scenario, the continental margin subducts to depths exceeding 100 km during the early stage of collision (Fig. 5a). Then, the sinking continental plate passes through the 410 km discontinuity and enters the mantle transition zone. This is mainly attributed to the positive Clapeyron slope of the 410 km phase transition, which increases the density of the subducting plate. Afterwards, the continental slab curves backward due to the resistance from the negative Clapeyron slope of the 660 km phase transition. Ultimately, while lithospheric necking is observed along the ocean-continent transition, complete slab breakoff remains inhibited (Fig. 5d).

## Discussion and conclusions

### Slab breakoff mode selection

**Fig. 6:**
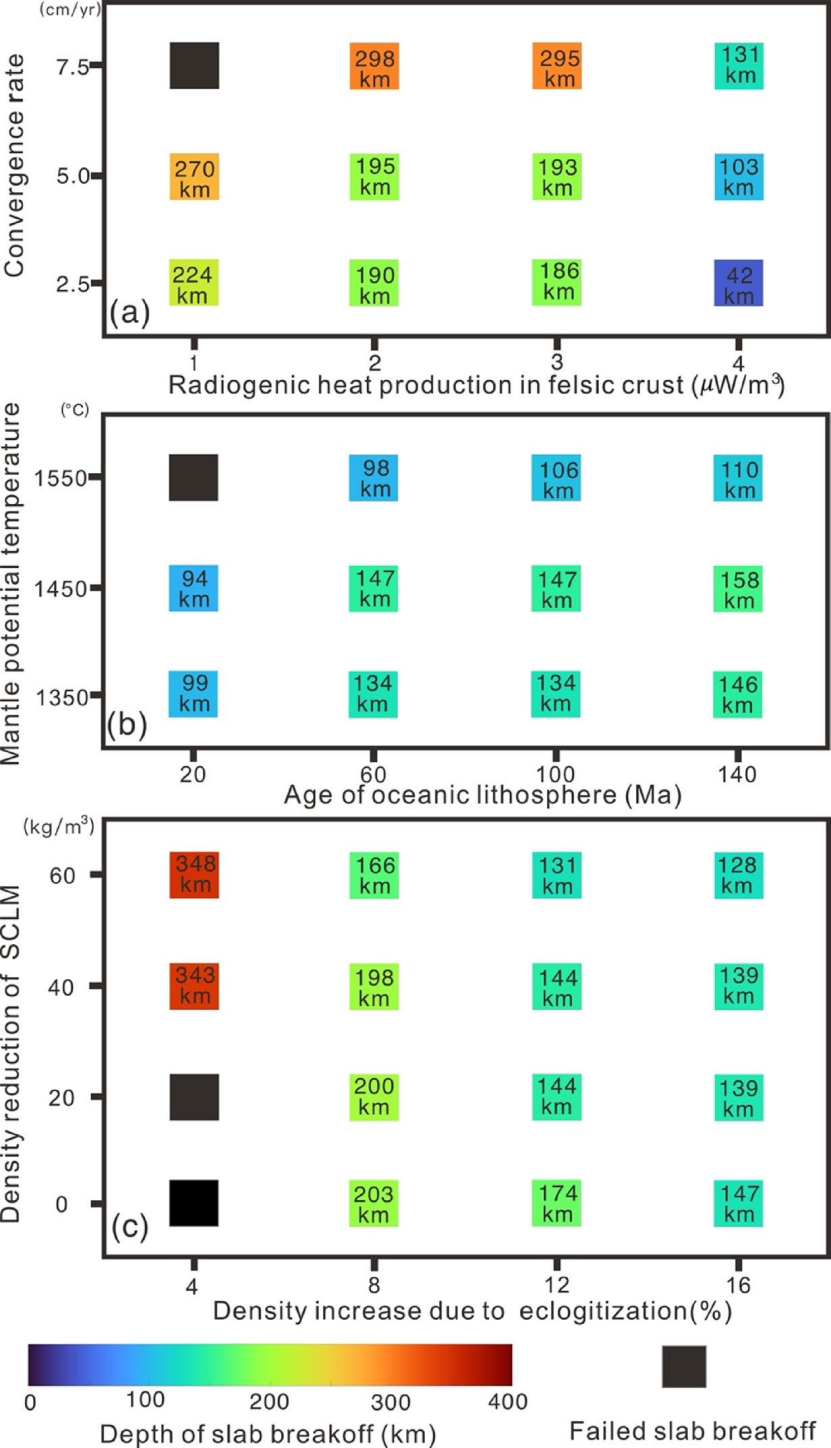
Description of the results of the parametric study. Discrimination of different depths of breakoff for each experiment. SCLM is subcontinental lithospheric mantle.

Our numerical models reveal three basic modes of slab breakoff in collisional orogens. Based on these results, we constructed regime diagrams (Fig. 6) to illustrate how the slab breakoff mode is governed by the convergence rate, the radiogenic heat production of felsic crust, the mantle potential temperature, the age of oceanic lithosphere, the density reduction of subcontinental lithospheric mantle (SCLM), and eclogitization-induced densification of oceanic crust.

Andrews and Billen^19^ have demonstrated the critical role of rheology in controlling slab breakoff dynamics. In particular, the strength of oceanic-continental transition zone plays a significant role, which is controlled by the thermal structures of both continental and oceanic lithosphere. For continental lithosphere, the temperature structure is controlled by the radiogenic heat production within felsic crust^32^. Higher radiogenic heat production corresponds to a hotter, weaker lithosphere. Under such conditions, slab breakoff occurs at shallower depths (Fig. 6a), with a minimum breakoff depth of 40 km in the model with an initially hot continental lithosphere (~ 4 μW/m^3^). Thus, sufficiently high crustal radiogenic heat production serves as a prerequisite for shallow slab breakoff. For oceanic lithosphere, the thermal structure is predominantly governed by mantle potential temperature (*T*_*P*_) and age of oceanic lithosphere. The regime diagram shows that deep slab breakoff is favored by older oceanic lithosphere and lower mantle potential temperature (Fig. 6b).

In addition, it is widely accepted that convective mantle traction and active hot mantle plumes can drive continental lithosphere motions in the modern Earth^39^. In our models, the continental plate continues to subduct under imposed convergence rates after oceanic subduction. A strong correlation between the convergence rates and the depth of breakoff was observed in our results. The higher continental convergence rates promote deeper slab breakoff rather than shallower slab breakoff. Based on the numerical results, the promoting factors of shallow slab breakoff include lower convergence rates and higher radiogenic heat production of continental crust.

Many forces act on the ocean-continent transition zone of subducting slab^40^. The overall effects of these forces control the dynamics as well as the mode selection of slab breakoff depth. The forces involved in the ocean-continent transition zone include a negative buoyancy force from oceanic plate, a positive buoyancy from continent plate, resistances from the plate and surrounding mantle, as well as the phase transition induced forces, e.g., eclogitization of oceanic crust^41^. However, the resistance is influenced by factors such as the slab interface friction coefficient and mantle viscosity, making it difficult to estimate variations in resistance. We assume that the resistance of the subducting slab remains constant in our models. Thus, the magnitude of the extensional forces in the ocean-continent transition zone depends on the buoyancy difference between oceanic and continental lithosphere. Compositional variations affect the density of the subcontinental lithosphere mantle (SCLM), with a higher degree of mantle depletion leading to lower densities^42^. Furthermore, the oceanic crust may undergo incomplete eclogitization due to the heterogeneous water content and the degree of phase transition^43,44^. In our model, variations in SCLM density and eclogitization-induced densification result in slab breakoff depths ranging from 146 to 366 km (Table S3). The results further suggest that the density increase associated with eclogitization exerts the dominant control on breakoff depth, while the influence of SCLM density reduction is relatively minor (Fig. 6c).

### Geological implications

While these idealized numerical models cannot fully capture the multiscale complexities of collisional orogens, their first-order results may offer useful insights into breakoff dynamics. An unresolved question is the absence of ultrahigh-pressure metamorphic (UHP) complexes in Archean orogens. The higher ambient upper-mantle temperatures of the ancient Earth or continental margin architecture may provide explanation for this observation^45^. However, our results provide a new explanation for the late appearance of UHP metamorphic rocks. Our results emphasize the thermo-mechanical role of radiogenic heating within the felsic crust and eclogitization of oceanic crust in controlling slab breakoff depth. Abundant geochemical studies have indicated that the Moho temperature of the Archean crust was higher than 700 ℃ at 3.5 Ga^29-31^. In addition, A hotter Archean mantle necessarily results in deeper and more voluminous peridotite melting during adiabatic decompression, which is expected to produce thicker oceanic crust^46,47^. Previous numerical modeling indicates that oceanic lithosphere derived from hotter mantle has greater negative buoyancy due to eclogitization of oceanic crust^48^. Consequently, combined with our results from numerical modeling, we infer that shallow breakoff represents the most characteristic form of lithospheric convergence occurring within the Archean orogens. As a classic example for understanding the evolution of Archean orogens, the Sino-Korea Craton (NCC) is the largest Archean block in East Asia. It was formed through the amalgamation of several small Archean blocks at ~1.85 Ga^49^ or 2.5 Ga^50^ along the Trans-North China Orogen. The NCC consists of granulite with high geothermal gradient and the widespread development of migmatites and granites with high radiogenic heat production^51^.The metamorphic evolution of NCC is dominated by anticlockwise type mostly involving isobaric cooling (IBC)^49^. Such IBC-type anticlockwise P-T paths generally reflect metamorphism related to the intrusion and underplating of large voluminous mafic magmas^52^, supporting the view that Archean orogens were dominated by shallow slab breakoff.

The Neo-Tethys tectonic system is a large orogenic belt located on the southern margin of the Eurasian continent, formed by progressive collision of Cimmerian continent rifted from the northern margin of the Gondwana^53^. The late Carboniferous to early Permian strata on Cimmeria are all characterized by thick limestone interlayered with sandstone and shale^54^, indicative of a passive continental margin environment with lower radiogenic heat production. In addition, some ultra-high-pressure metamorphic rocks imply that continental crust could subduct to depths of 100-400 km in the Alps and Himalayan orogens^55^. Taken together with the sedimentary successions and exhumation of ultra-high-pressure continental slivers, the evidence supports the subduction with deep slab breakoff in Phanerozoic orogens. Seismic imaging could provide important evidence for slab breakoff beneath orogens. Previous studies reveal continental crust underwent deep subduction, dragged by the preceding oceanic slab, with a low-velocity anomaly between 110 and 150 km beneath the Western Alps7.Similar seismic tomography evidence is observed in the Pamir-Hindu-Kush region, located at the western Himalayan Syntaxis^56^. The seismic velocity anomaly shows that slab breakoff occurs at 250–300 km and that intermediate depth earthquakes cluster at the neck connecting it to the deeper slab, providing a rare glimpse of the ephemeral process of deep slab breakoff^57^.

## Supplementary Information

Below is the link to the electronic supplementary material.

Supplementary Material 1

## Data Availability

All the relevant data and model output presented in this study are available from the corresponding author upon reasonable request.

## Code availability

The numerical modeling code that supports the finding of this study is available from the corresponding author upon reasonable request.
