# Supplementary material for: Depth of slab breakoff in Archean: the role of radiogenic heat production of continental crust and eclogitization of oceanic crust
Source: Sci Rep. 2026 Jan 3;16:35. doi: 10.1038/s41598-025-29212-x (PMC12764590; doi:10.1038/s41598-025-29212-x)
Supplement: Supplementary file 1 — Supplementary Material 1 [file 41598_2025_29212_MOESM1_ESM.docx]

Supplementary information for

**Depth of slab breakoff in Archean: the role of radiogenic heat production of continental crust and eclogitization of oceanic crust**

**Yin-Long Fan^1^, Li-Fei Zhang^1*^, Yang Wang^2^, Zhong-Hai Li^2^**

*^1^SKLab-DeepMinE, MOEKLab-OBCE, School of Earth and Space Sciences, Peking University, Beijing, China,*

*^2^State Key Laboratory of Earth System Numerical Modeling and Application, College of Earth and Planetary Sciences, University of Chinese Academy of Sciences, Beijing, China*

*Correspondence to: L.-F Zhang, lfzhang@pku.edu.cn

## 1.Numerical Methodology

## 1.1. Governing Equations

The 2-D Stokes equation for conservation of momentum is given by:

| $\frac{\text{∂σ'}_{\text{ij}}}{\text{∂x}_{\text{j}}}\text{-}\frac{\text{∂P}}{\text{∂x}_{\text{i}}}\text{=}\text{g}_{\text{i}}\text{ρ}\text{(}\text{C, M ,P ,T}\text{)}$ | (1) |
| --- | --- |

where *i* and *j* represent spatial direction following the Einstein summation convention; *x_i_* is the spatial coordinate; $\text{σ'}_{\text{ij}}$ is the deviatoric stress tensor; *g_i_* is the gravitational acceleration; and the density *ρ* depends on pressure (*P*), temperature (*T*), composition (*C*), and melt fraction (*M*).

Conservation of mass is approximated by the incompressible continuity equation:

| $\frac{\text{∂v}_{\text{i}}}{\text{∂x}_{\text{i}}}\text{=0}$ | (2) |
| --- | --- |

where *v_i_* is the component of the velocity vector.

The heat conservation equation is shown as:

| $\text{ρ}\text{C}_{\text{p}}\frac{\text{DT}}{\text{Dt}}\text{=-}\frac{\text{∂}\text{q}_{\text{i}}}{\text{∂}\text{x}_{\text{i}}}\text{+}\text{H}_{\text{r}}\text{+}\text{H}_{\text{a}}\text{+}\text{H}_{\text{s}}\text{+}\text{H}_{\text{L}}$  $\text{H}_{\text{a}}\text{=Tα}\frac{\text{DP}}{\text{Dt}}$  $\text{H}_{\text{s}}\text{=}\text{σ'}_{\text{ij}}\dot{\text{ε}_{\text{ij}}}$  $\text{q}_{\text{i}}\text{=-k}\text{(}\text{T,P,C}\text{)}\frac{\text{∂T}}{\text{∂}\text{x}_{\text{i}}}$ | (3) |
| --- | --- |

where *DT/Dt* is the full derivative of the temperature with respect to time; *C_p_* is the heat capacity; *q_i_* is the heat flux; *H* represents the sum of individual heat sources (e.g., radioactive heat production, mechanical/shear heating, adiabatic heating, and latent heat); and *k* is the thermal conductivity as a function of composition, temperature, and pressure.

The constitutive relationship is following:

| $\text{ σ'}_{\text{ij}}\text{=2}\text{η}_{\text{eff}}{\dot{\text{ε}}}_{\text{ij}}$  $\dot{\varepsilon}_{ij}\text{=}\frac{\text{1}}{\text{2}}\left( \frac{\text{∂}\text{v}_{\text{i}}}{\text{∂}x_{j}}\text{+}\frac{\text{∂}\text{v}_{\text{j}}}{\text{∂}x_{i}} \right)$ | (4) |
| --- | --- |

## 1.2. Rheological Model

The rheology in this study is described by visco-plastic flow law as a combination of ductile creep and Mohr-Coulomb yield criterion. Viscosities for creep as a function of pressure, temperature, composition and strain rate invariant are computed as follows:

| $\text{η}_{\text{ductile}}\text{=}\left( {\dot{\text{ε}}}_{\text{Ⅱ}} \right)^{\frac{\text{1-n}}{\text{n}}}\text{F}\left( \text{A}_{\text{D}} \right)^{\text{-}\frac{\text{1}}{\text{n}}}\text{exp}\left( \frac{\text{E+PV}}{\text{nRT}} \right)$ | (5) |
| --- | --- |

where ${\dot{\text{ε}}}_{\text{Ⅱ}}$ is the second invariant of strain rate tensor; and *A_D_*, *E*, *V* and *n* are experimentally determined flow law parameters (Table 1), which stand for material constant, activation energy, activation volume and stress exponent, respectively. *F* is a dimensionless coefficient depending on the type of experiments on which the flow law is based.

The plastic rheology with the Mohr-Coulomb yield criterion is implemented as follows:

| $\text{σ}_{\text{yield}}\text{=}\text{C}_{\text{0}}\text{+P}\sin\text{(}\text{φ}_{\text{eff}}\text{)}$  $\text{η}_{\text{plastic}}\text{=}\frac{\text{σ}_{\text{yield}}}{\text{2}{\dot{\text{ε}}}_{\text{Ⅱ}}}$ | (6) |
| --- | --- |

where $\text{σ}_{\text{yield}}$ is the yield stress; $\text{C}_{\text{0}}$ is the cohesion; $\text{φ}_{\text{eff}}$ is the effective internal frictional angle.

The final visco-plastic rheology is determined by comparing brittle/plastic and ductile viscosities as a function of depth, which is further controlled by the cut-off values of [10^18^,10^26^] Pa s

| $\text{η}_{\text{creeep}}\text{=min}\left( \text{η}_{\text{ductile}}\text{, }\text{η}_{\text{plastic}} \right)$ | (7) |
| --- | --- |

## 1.3 density model

We use the extended Boussinesq approximation with the incompressible continuity equation and variable density in the momentum and energy conservation equations. The density of rocks varies with pressure (P) and temperature (T) according to the equation:
${\text{ }\text{ρ}}_{\text{solid}}\text{=}\text{ρ}_{\text{0}}\left[ \text{1-α}\left( \text{T-}\text{T}_{\text{0}} \right) \right]\left[ \text{1+β}\left( \text{P-}\text{P}_{\text{0}} \right) \right]$(8)

where ρ_0_ is the standard density at P_0_ = 1 MPa and T_0_ = 298 K, and α = 1 × 10^−5^ Κ^−1^ and β = 3 × 10^−12^ Pa^−1^ are the coefficients of thermal expansion and compressibility, respectively. Our models take into account the phase transformations of olivine into wadsleite and ringwoodite and into bridgmanite in the mantle (Ito et al., 1990; Katsura and Ito, 1989). Eclogitization of subducted basaltic crust is taken into account by linearly increasing the density of the crust with pressure from 0% to 16% in the P–T region between the experimentally determined garnet-in and plagioclase-out phase transitions in basalt (Ito and Kennedy, 1971).

## 1.4. Topography

The top surface is calculated dynamically as a free surface using an 18-22 km thick top layer with a low viscosity (10^18^ Pa s) above the felsic crust. The composition is either “air” (1 kg/m^3^, above z=10 km water level) or “water” (1000 kg/m^3^, below z=10 km water level). The interface between this layer and the top of the oceanic/continental crust is treated as an internal erosion/sedimentation surface, which evolves according to the transport equation solved at each time-step (Gerya and Yuen, 2003). The equation is expressed as follows:

| $\frac{\text{∂}\text{y}_{\text{es}}}{\text{∂t}}\text{=}\text{v}_{y}\text{-}\text{v}_{\text{x}}\frac{\text{∂}y_{\text{es}}}{\text{∂x}}\text{-}\text{v}_{\text{s}}\text{+}\text{v}_{\text{e}}$ | (9) |
| --- | --- |

where *y_es_* is the vertical position of the surface as a function of the horizontal distance *x*; *v_s_* and *v_e_* are the sedimentation and erosion rates, respectively, calculated by:

*v_s_=*0 *mm/yr, v_e_=v_e0_, when: y_es_<*18 *km*

*v_s_=v_s0_, v_e_=*0 *mm/yr, when: y_es_>*22 *km*

where *v_e0_* and *v_s0_* are the imposed constant large-scale erosion and sedimentation rates, respectively.

## 2.Supplementary Table

**Table S1 Material properties used in the numerical experiments**

| Material | *ρ_0_* | *η_eff_^a^* | *φ_0_* | *H_r_* | *k_0_^c^* | *k_1_^c^* |
| --- | --- | --- | --- | --- | --- | --- |
|  | Kg/m^3^ | Flow law |  | μW m^-3^ | W m^-1^ K^-1^ | W m^-1^ |
| Air | 1 | A | 0 | - | 200 | 0 |
| Water | 1000 | A | 0 | - | 200 | 0 |
| Felsic continental crust | 2700 | B | 0.15 | 2.0 | 0.64 | 807 |
| Mafic continental crust | 2900 | C | 0.15 | 0.5 | 1.18 | 474 |
| Oceanic crust | 3000 | C | 0.60 | 0.5 | 1.18 | 474 |
| Oceanic lithospheric mantle | 3370 | D | 0.6 | 0.022 | 0.73 | 1293 |
| Pro-continental lithospheric mantle | Varying | D | 0.6 | 0.022 | 0.73 | 1293 |
| Pro-continental lithospheric mantle | 3370 | D | 0.50 | 0.022 | 0.73 | 1293 |
| Oceanic lithospheric mantle | 3370 | D | 0.30 | 0.022 | 0.73 | 1293 |
| Asthenospheric mantle | 3370 | D | 0.26 | 0.022 | 0.73 | 1293 |
| Weak zone | 3200 | E | 0.006 | 0.022 | 0.73 | 1293 |
| References | 1,2 | 4 | - | 1 | 3 | 3 |

*ρ_0_*, *k*, *Hr*, *C_p_* and *φ_0_* are the reference density, thermal conductivity, radiogenic heat production, heat capacity at constant pressure and effective internal frictional angle used for plasticity, respectively.

^a^ Parameters of viscous flow laws are shown in Tables 2.

^c^ *k*=[*k_0_*+*k_1_*/(T+77)]exp(0.00004P).

References 1-4 are Turcotte et al. (2002); Bittner and Schmeling (1995); Clauser and Huenges (1995); Ranalli (1995), respectively.

**Table S2 Viscous flow laws used in the numerical experiments ^a^**

| ID symbol | Flow laws | E | V | n | A_D_ | η_0_^b^ |
| --- | --- | --- | --- | --- | --- | --- |
|  |  | (KJ mol^-1^) | (J MPa^-1^mol^-1^) |  | (MPa^-n^ s^-1^) | (Pa^n^ s) |
| A | Air/water | 0 | 0 | 1.0 | 1.0×10^-12^ | 1.0×10^18^ |
| B | Wet quartzite | 154 | 0 | 2.3 | 3.2×10^-4^ | 1.97×10^17^ |
| C | Plagioclase An_75_ | 238 | 0 | 3.2 | 3.3×10^-4^ | 4.80×10^22^ |
| D | Dry olivine | 532 | 8 | 3.5 | 2.5×10^4^ | 3.98×10^16^ |
| E | Wet olivine | 470 | 8 | 4.0 | 2.0×10^3^ | 5.01×10^20^ |

^a^ E, V, n, A_D_ and η_0_ are the activation energy, activation volume, stress exponent, material constant, and pre-exponential viscous factor, respectively.

^b^ η_0_ is the reference viscosity, which is calculated: η_0_ = (1/A_D_)×10^6n^.

References: Kirby (1983); Kirby and Kronenberg (1987); Ranalli and Murphy (1987); Ji and Zhao (1993); Ranalli (1995).

**Table S3 Parameters and breakoff information for all models within the parametric study. SCLM represents subcontinental lithospheric mantle**

| Model | Convergence  Rate  (cm/yr) | Mantle potential  temperature  (°C) | | Density reduction  of SCLM  (kg/m^3^) | | Radiogenic heat production in felsic crust  (μW/m^3^) | Age of oceanic lithosphere  (Ma) | Density increasing of oceanic crust eclogitization  (%) | Depth of slab breakoff  (km) |
| --- | --- | --- | --- | --- | --- | --- | --- | --- | --- |
| 1 | 5 | 1350 | 0 | | 2 | | 60 | 4 | Failed |
| 2 | 5 | 1350 | 0 | | 2 | | 60 | 8 | 195 |
| 3 | 5 | 1350 | 0 | | 2 | | 60 | 12 | 174 |
| 4 | 5 | 1350 | 0 | | 2 | | 60 | 16 | 134 |
| 5 | 5 | 1350 | 20 | | 2 | | 60 | 4 | Failed |
| 6 | 5 | 1350 | 20 | | 2 | | 60 | 8 | 200 |
| 7 | 5 | 1350 | 20 | | 2 | | 60 | 12 | 144 |
| 8 | 5 | 1350 | 20 | | 2 | | 60 | 16 | 139 |
| 9 | 5 | 1350 | 40 | | 2 | | 60 | 4 | 343 |
| 10 | 5 | 1350 | 40 | | 2 | | 60 | 8 | 198 |
| 11 | 5 | 1350 | 40 | | 2 | | 60 | 12 | 144 |
| 12 | 5 | 1350 | 40 | | 2 | | 60 | 16 | 139 |
| 13 | 5 | 1350 | 60 | | 2 | | 60 | 4 | 348 |
| 14 | 5 | 1350 | 60 | | 2 | | 60 | 8 | 166 |
| 15 | 5 | 1350 | 60 | | 2 | | 60 | 12 | 131 |
| 16 | 5 | 1350 | 60 | | 2 | | 60 | 16 | 128 |
| 17 | 2.5 | 1350 | 0 | | 1 | | 60 | 8 | 224 |
| 18 | 2.5 | 1350 | 0 | | 2 | | 60 | 8 | 190 |
| 19 | 2.5 | 1350 | 0 | | 3 | | 60 | 8 | 186 |
| 20 | 2.5 | 1350 | 0 | | 4 | | 60 | 8 | 42 |
| 21 | 5 | 1350 | 0 | | 1 | | 60 | 8 | 270 |
| 22 | 5 | 1350 | 0 | | 2 | | 60 | 8 | 195 |
| 23 | 5 | 1350 | 0 | | 3 | | 60 | 8 | 193 |
| 24 | 5 | 1350 | 0 | | 4 | | 60 | 8 | 103 |
| 25 | 7.5 | 1350 | 0 | | 1 | | 60 | 8 | Failed |
| 26 | 7.5 | 1350 | 0 | | 2 | | 60 | 8 | 298 |
| 27 | 7.5 | 1350 | 0 | | 3 | | 60 | 8 | 295 |
| 28 | 7.5 | 1350 | 0 | | 4 | | 60 | 8 | 131 |
| 29 | 5 | 1350 | 0 | | 2 | | 20 | 16 | 99 |
| 30 | 5 | 1350 | 0 | | 2 | | 60 | 16 | 134 |
| 31 | 5 | 1350 | 0 | | 2 | | 100 | 16 | 134 |
| 32 | 5 | 1350 | 0 | | 2 | | 140 | 16 | 146 |
| 33 | 5 | 1450 | 0 | | 2 | | 20 | 16 | 94 |
| 34 | 5 | 1450 | 0 | | 2 | | 60 | 16 | 147 |
| 35 | 5 | 1450 | 0 | | 2 | | 100 | 16 | 147 |
| 36 | 5 | 1450 | 0 | | 2 | | 140 | 16 | 158 |
| 37 | 5 | 1550 | 0 | | 2 | | 20 | 16 | Failed |
| 38 | 5 | 1550 | 0 | | 2 | | 60 | 16 | 98 |
| 39 | 5 | 1550 | 0 | | 2 | | 100 | 16 | 106 |
| 40 | 5 | 1550 | 0 | | 2 | | 140 | 16 | 110 |

## Reference

Bittner, D., Schmeling, H., (1995). Numerical Modelling of Melting Processes and Induced Diapirism In the Lower Crust. Geophysical Journal International, 123, 59-70.

Clauser, C., Huenges, E., 1995. Thermal Conductivity of Rocks and Minerals, Rock Physics & Phase Relations, pp. 105-126.

Gerya, T.V., Yuen, D.A., (2003). Rayleigh–Taylor instabilities from hydration and melting propel ‘cold plumes’ at subduction zones. Earth and Planetary Science Letters, 212, 47-62.

Ito, K., Kennedy, G.C., 1971. An Experimental Study of the Basalt-Garnet Granulite-Eclogite Transition, The Structure and Physical Properties of the Earth's Crust, pp. 303-314.

Ito, E., Akaogi, M., Topor, L., Navrotsky, A., 1990. Negative Pressure-Temperature Slopes for Reactions Formign MgSiO_3_ Perovskite from Calorimetry. Science 249, 1275-1278.

Katsura, T., Ito, E., 1989. The system Mg_2_SiO_4_-Fe_2_SiO_4_ at high pressures and temperatures: Precise determination of stabilities of olivine, modified spinel, and spinel. Journal of Geophysical Research: Solid Earth 94, 15663-15670.

Kirby, S.H., 1983. Rheology of the lithosphere. Rev. Geophys. 21, 1458-1487.

Kirby, S.H., Kronenberg, A.K., 1987. Rheology of the lithosphere: Selected topics. Rev. Geophys. 25, 1219-1244.

Ranalli, G., Murphy, D.C., 1987. Rheological stratification of the lithosphere. Tectonophysics, 132, 281-295.

Ji, S., Zhao, P., 1993. Flow laws of multiphase rocks calculated from experimental data on the constituent phases. Earth and Planetary Science Letters. 117, 181-187.

Ranalli, G., 1995. Rheology of the Earth. Chapman & Hall.

Schmidt, M.W., Poli, S., 1998. Experimentally based water budgets for dehydrating slabs and consequences for arc magma generation. Earth and Planetary Science Letters, 163, 361-379.

Turcotte, D.L., Schubert, G., Schubert, J., 2002. Geodynamics. Cambridge University Press.
